# Training experience is an important factor affecting willingness for bystander CPR and awareness of AED: a survey of residents from a province in Central China in 2023

**DOI:** 10.3389/fpubh.2024.1459590

**Published:** 2024-09-02

**Authors:** Xueli Tian, Yongle Zhang, Dongmei Dou

**Affiliations:** ^1^Department of Orthopaedics, Huaihe Hospital, Henan University, Kaifeng, China; ^2^School of Nursing and Health, Henan University, Kaifeng, China

**Keywords:** out-of-hospital cardiac arrest, cardiopulmonary resuscitation, automated external defibrillators, training experience, questionnaire survey

## Abstract

**Background:**

Bystander cardiopulmonary resuscitation (CPR) and the use of automated external defibrillators (AEDs) may improve survival in patients with out-of-hospital cardiac arrest (OHCA). The purpose of this study was to investigate the effect of CPR training experience and sociodemographic characteristics on bystander CPR willingness and AED awareness.

**Methods:**

In this study, a questionnaire survey was conducted among 3,569 residents in central China. Descriptive statistics, multiple linear regression and multivariate logistic regression modeling were used to investigate the effect of training experience and sociodemographic characteristics on knowledge of cardiac arrest first aid, awareness of AEDs, and willingness for bystander CPR.

**Results:**

Of the 3,569 participants, nearly 52% were female, 69.6% were < 23 years old, 23.5% had CPR training and 22.1% had witnessed OHCA. Characteristics of increasing bystander CPR willingness included CPR training experience, male, witnessed OHCA but not acting, knowing whether family members have cardiac disease, older age (>40 years) and lower level of education. Farmers were the subgroup with the least awareness of AED and knowledge of first aid.

**Conclusion:**

In China, CPR training experience was an important factor in improving bystanders’ CPR willingness, AED awareness and knowledge of cardiac arrest first aid. Additionally, having witnessed OHCA also had a positive effect on bystander CPR willingness.

## Introduction

Cardiac arrest (CA) is a sudden loss of blood flow caused by a sudden stop in the heart, which often leads to death if left untreated within minutes (1). Out-of-hospital cardiac arrest (OHCA) is a common time-critical disease that occurs in the out-of-hospital environment (2) and is also a major public health problem that causes a large number of deaths worldwide (3). The most common OHCA occurs in the patient’s home, and the patient’s family members often witness the occurrence of the event (4). According to observational studies, 5 million people worldwide suffer from OHCA every year, and only 7% of them survive (5, 6). Therefore, an increasing number of scholars have begun to focus on how to improve the survival rate of patients with OHCA.

Bystander cardiopulmonary resuscitation (CPR) is one of the key links in the survival chain of OHCA patients, nearly doubling the chances of survival (1, 7–9). Moreover, studies have shown that bystander CPR not only increases the survival chances of OHCA patients (7–10) but also has a positive impact on the prognosis of patients with good neurological status (11, 12). Therefore, many organizations that focus on improving survival advocate the use of extensive community education and training initiatives to increase bystander CPR rates and OHCA survival rates (3, 13). In addition, the use of automated external defibrillators (AED) also has a positive impact on the survival of patients in OHCA (14–16).

However, the initiation of CPR and AED is not optimal (14, 17, 18). In Europe, medical students need to not only improve their knowledge about CA and CPR but also incorporate AED training into mandatory courses (19). In addition, Asians have lower AED usage than white participants (20). Therefore, scholars from various countries have begun to explore how to improve the CPR rate and AED utilization rate in various countries through more effective training (14, 21).

To increase the prevalence of bystander CPR, intervention targets need to be identified (22). However, limited research has been done on the relationship between the type of bystander witness and bystander CPR initiation (22). A recent study in Singapore (22) and Japan (23) demonstrated that the witness type was associated with CPR initiation and OHCA outcomes. In China, little is known about the characteristics of those trained or willing to act when witnessing an OHCA. However, such information is useful to training providers, policy makers, and researchers for identifying coverage of training campaigns (14).

Therefore, the purpose of this study was to investigate the impact of sociodemographic characteristics, CPR training and witnessed OHCA on willingness for bystander CPR, first aid knowledge scores for CA, and awareness of AEDs. Based on this, the characteristics of bystanders’ CPR willingness were explored to further effectively improve the survival rate of OHCA patients.

## Methods

### Study design

This is a cross-sectional study on the willingness for bystander CPR, AED awareness and CA first aid knowledge among residents in a province in central China. This study was conducted from May to July 2023 and approved by the Biomedical Research Ethics Subcommittee of Henan University (HUSOM2023-457). Informed consent was obtained from the respondents. In this study, 3,800 online questionnaires were randomly distributed to residents of a central province by using social media, and 3,569 qualified questionnaires were finally collected.

### Data collection and variables

The inclusion criteria of the respondents were as follows: (1) residents of a province in central China and (2) independently completed the questionnaire. The exclusion criteria were as follows: (1) medical staff and (2) incomplete content. The content of the questionnaire was from the mature questionnaire-*Investigation on public knowledge and attitude toward first aid for cardiac arrest* (24), and the author’s consent was obtained. The questionnaire consisted of three parts: social demographic characteristics, cardiac arrest first aid knowledge, first aid attitude and training needs. Questionnaires were distributed and recovered to the respondents through the Questionnaire Star platform. After collecting the questionnaire, the data were exported in Excel form, collated and analyzed by two people after checking and confirming that the content was correct.

Bystander CPR was defined as CPR attempted by a bystander prior to arrival of the emergency medical services team, which included both telephone dispatcher-assisted CPR and bystander-initiated CPR (22). Bystander CPR willingness was defined as the respondents’ self-reported willingness to perform CPR when witnessing an OHCA. In addition, the CA first aid basic knowledge included identifying the most common place of cardiac arrest and the recognition of cardiac arrest. The CA first aid operational knowledge included five questions: phone 120 when CA occurs, chest compressions position, chest compressions frequency, chest compressions depth, and chest compressions importance. The questions of cardiac arrest first aid knowledge were multiple choice questions, with only one correct option, and the other three were all wrong options. For each correct answer, one point was scored, and the wrong answer was not scored. Therefore, the CA first aid basic knowledge was full of 2, the CA first aid operational knowledge was full of 5, and the total CA first aid knowledge was full of 7. The basic mastery of cardiac arrest emergency operational knowledge was defined as the score of CA first aid operational knowledge ≥3 and basic mastery of first aid knowledge of cardiac arrest was defined as the total score of CA first aid knowledge ≥5 (24).

Sociodemographic characteristics included sex, age, level of education, occupation, whether they were family members of cardiac patients and whether they had witnessed OHCA. ‘Yes, and acting’ was defined as having participated in bystander CPR when witnessing OHCA; ‘yes, but not acting’ was defined as not participating in bystander CPR when witnessing OHCA. The sex of the respondents was categorically defined as male and female. Age was grouped into one of three categories: below 23 years old, 23–40 years old, and over 40 years old. The educational level was classified as high school or below, universities (including junior colleges), and graduate degree or above. Occupation was classified as school students, employees of enterprises and institutions, workers, farmers, and others (self-employment and retirement).

### Statistical analysis

Descriptive analysis was conducted to examine the distribution of categorical and continuous variables. The categorical variables were presented as numbers and percentages. Normality of the continuous variables was assessed using the Shapiro–Wilk test, and variables that conformed to a normal distribution were presented as mean and standard deviation, while non-normally distributed variables were presented as median and interquartile range. In this study, the continuous variables that conform to the normal distribution were presented as mean and standard deviation. For 11 categorical variables, Pearson’s chi-square test was used to assess the frequency of sociodemographic characteristics. For the three quantitative data points of the CA first aid basic knowledge score, CA first aid operational knowledge score and CA first aid knowledge total score, ANOVA was used to assess the concentration trend of sociodemographic characteristics. In addition, multiple linear regression was used to analyze the association between the CA first aid knowledge score and sociodemographic characteristics. Moreover, logistic regression analysis was used to evaluate the association between AED awareness, bystander CPR willingness and sociodemographic characteristics. SPSS 25.0 software was used for statistical analysis, and a *p* value <0.05 was used to determine statistical significance.

## Results

Table 1 presents the characteristics of 3,569 participants. The response rate of this study was 93.9%, and 231 questionnaires were excluded due to incomplete content. More than half were female (51.9%), nearly 70% of the participants were < 23 years old (69.6%), and most were school students (67.6%). Most had education at the university level (75.4%). A total of 17.5% of the participants indicated that they were family members of cardiac patients, 6.3% had witnessed OHCA and acting, and 15.8% had witnessed OHCA but not acting. In addition, the table also shows the demographic characteristics of participants (23.5%) who had experienced CPR training. Significantly more male, participants who witnessed OHCA and acting, those aged 23–40, graduate students and above, and family members of cardiac patients reported that they had participated in CPR training. There was no significant difference by occupation.

**Table 1:** Sociodemographic characteristics (*N* = 3,569).

Variables	Total, N(%)	Trained in cardiopulmonary resuscitation, N(%)	
		Yes	No
Total, N(%)	3,569(100.0)	838(23.5)	2,731(76.5)
Sex			
Male	1715(48.1)	456(26.6)	1,259(73.4)
Female	1854(51.9)	382(20.6)	1,472(79.4)
χ^2^ (*P value*)		17.761(<0.001)	
Age group, years			
<23	2,484(69.6)	604(24.3)	1880(75.7)
23–40	645(18.1)	169(26.2)	476(73.8)
>40	440(12.3)	65(14.8)	375(85.2)
χ^2^ (*P value*)		22.191(<0.001)	
Educational level			
High school or below	708(19.8)	135(19.1)	573(80.9)
Universities (including junior colleges)	2,691(75.4)	653(24.3)	2038(75.7)
Graduate degree or above	170(4.8)	50(29.4)	120(70.6)
χ^2^ (*P value*)		11.926(0.003)	
Occupation			
School students	2,414(67.6)	582(24.1)	1832(75.9)
Enterprises	384(10.8)	89(23.2)	295(76.8)
Workers	142(4.0)	34(23.9)	108(76.1)
Farmers	131(3.7)	23(17.6)	108(82.4)
Others (Self-employment and retirement)	498(13.9)	110(22.1)	388(77.9)
χ^2^ (*P value*)		3.663(0.453)	
Family members of cardiac patients			
Yes	625(17.5)	193(30.9)	432(69.1)
No	2,630(73.7)	579(22.0)	2051(78.0)
Do not sure	314(8.8)	66(21.0)	248(79.0)
χ^2^ (*P value*)		23.248(<0.001)	
Witnessed out-of-hospital cardiac arrest			
Yes, and acting	225(6.3)	114(50.7)	111(49.3)
Yes, but no acting	565(15.8)	266(47.1)	299(52.9)
No	2,779(77.9)	458(16.5)	2,321(83.5)
χ^2^ (*P value*)		343.474(<0.001)	

Most participants knew that cardiac arrest most often occurred outside the hospital, at home (39.0%) and in public places (39.6%). Among them, subjects aged 23–40 (41.4%, *p* < 0.001), employees of enterprises and institutions (45.1%, *p* < 0.001), and family members of cardiac patients (52.6%, *p* < 0.001) were more likely to find that the most common place of cardiac arrest was at home. More than half of the participants correctly recognized cardiac arrest (54.9%), and those with university education (including junior college) were more likely to correctly recognize cardiac arrest (57.0%, *p <* 0.001; Supplementary Table S1).

Among the five questions of phone, position, frequency, depth and importance, the subgroups with the highest accuracy rates were > 40 years of age, who witnessed CA but not acting, who trained in CPR, who witnessed CA and acting, and who trained in CPR (*p* < 0.05 for all; Supplementary Table S2).

**Figure 1:**
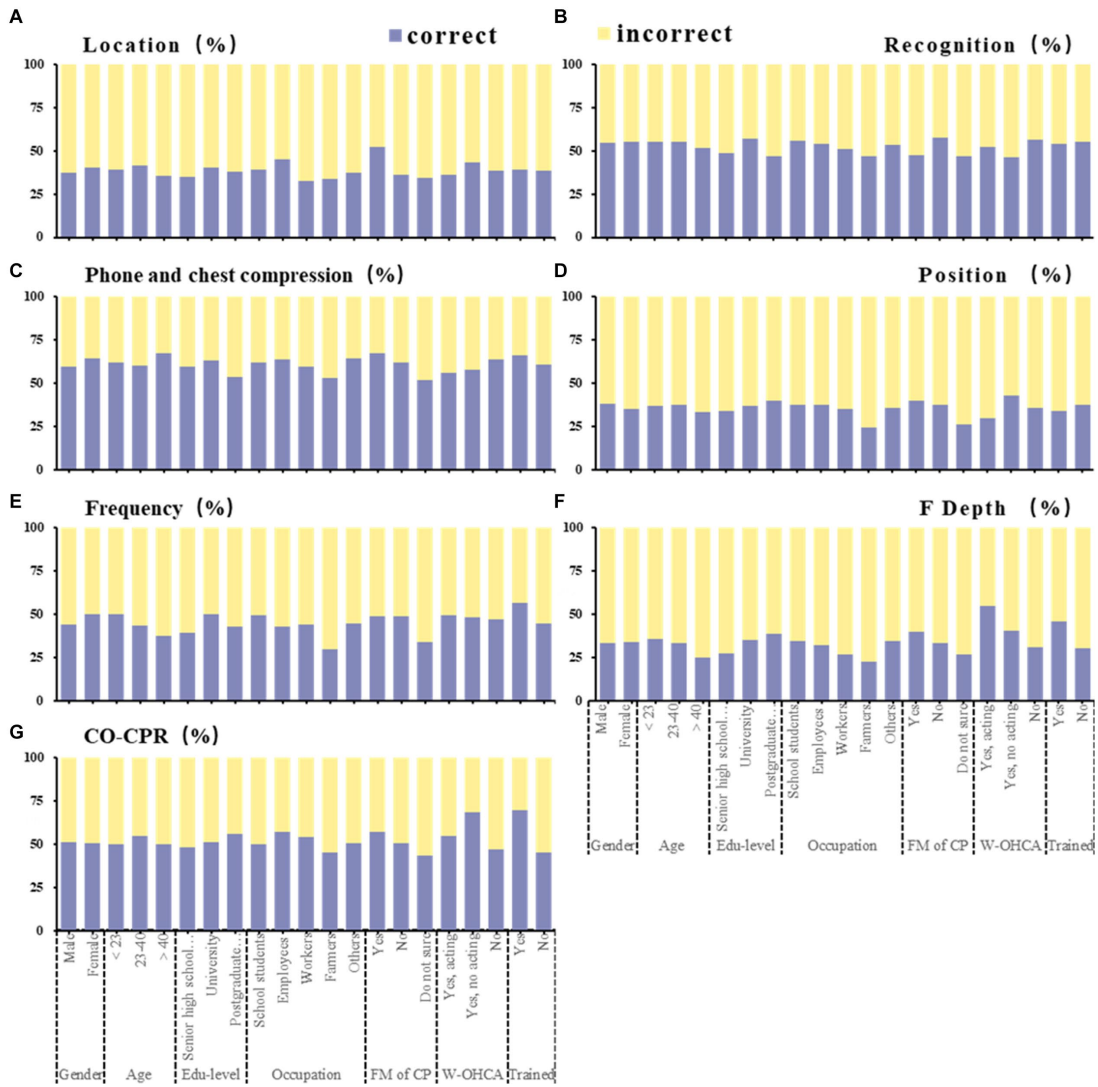
Seven categories of knowledge questions among participants (*N* = 3,569). This figure shows the proportion of correct and incorrect counts by sociodemographic characteristics in each question. Association of sociodemographic characteristics with proportion of the cardiac arrest most common location **(A)**, cardiac arrest recognition **(B)**, phone 120 and chest compression when cardiac arrest occurs **(C)**, position of chest compressions **(D)**, frequency of chest compressions **(E)**, depth of chest compressions **(F)**, importance of chest compression **(G)**. Correct location: At home; Correct recognition: Found that someone suddenly fainted, no response to his/her shouting; Correct phone: Phone 120 and chest compression; Correct position: Left chest; Correct frequency: 100–120 times/min; Correct depth: 5–6 cm; Correct importance: Only chest compressions can save lives. CO-CPR, only chest compressions; FM of CP, family member of cardiac patient; W-OHCA, witnessed out-of-hospital cardiac arrest.

Figure 1 shows that the highest accuracy rate is for phone 120 and start chest compressions (Figure 1C), followed by CA recognition (Figure 1B) and chest compression importance (Figure 1G; average above 50%). The accuracy of identifying the most common place of CA at home (Figure 1A), the chest compression location (Figure 1D), the chest compression frequency (Figure 1E), and the chest compression depth (Figure 1F) ranged from 25 to 50%.

**Figure 2:**
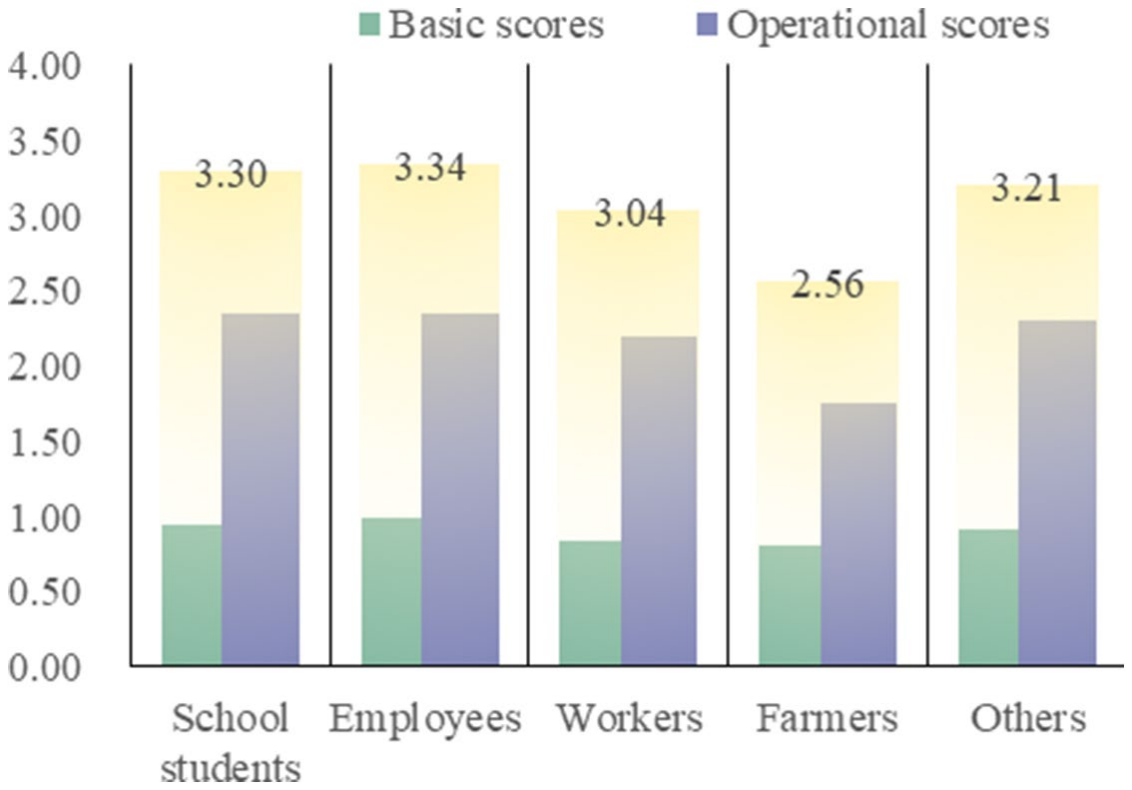
The scores of each group under the occupational category.

Overall, the average score of basic knowledge score and operational knowledge score is less than half, the subgroup with the highest basic knowledge score is 0.99 points (out of 2.00) for employees of enterprises and institutions, and the subgroup with the highest operational knowledge score and total score is 2.73 points (out of 5.00) and 3.67 points (out of 7.00) for personnel who have trained in CPR. The three lowest subgroups were all farmers, with knowledge scores of 0.81, 1.76 and 2.56 (*p* < 0.05 for all; Table 2). Figure 2 shows the scores of each group under the occupational category, with farmers showing significantly low scores.

**Table 2:** Cardiac arrest first aid knowledge score.

Variables	CA first aid basic knowledge score (2.00)		CA first aid operation knowledge score (5.00)		Total score for first aid knowledge of CA (7.00)	
	Mean ± SD	*P* value	Mean ± SD	*P* value	Mean ± SD	*P* value
Total	0.94 ± 0.70		2.31 ± 1.28		3.25 ± 1.56	
Sex		0.142		0.057		0.027
Male	0.92 ± 0.70		2.27 ± 1.27		3.19 ± 1.58	
Female	0.96 ± 0.70		2.35 ± 1.29		3.31 ± 1.55	
Age group, years		0.082		0.006		0.002
<23	0.94 ± 0.70		2.35 ± 1.29		3.29 ± 1.57	
23–40	0.97 ± 0.69		2.30 ± 1.28		3.27 ± 1.53	
>40	0.87 ± 0.72		2.14 ± 1.24		3.01 ± 1.58	
Educational level		<0.001		<0.001		<0.001
High school or below	0.83 ± 0.71		2.09 ± 1.22		2.93 ± 1.53	
Universities (including junior colleges)	0.97 ± 0.70		2.37 ± 1.28		3.34 ± 1.55	
Graduate degree or above	0.85 ± 0.66		2.31 ± 1.43		3.16 ± 1.67	
Occupation		0.028		<0.001		<0.001
School students	0.95 ± 0.70		2.35 ± 1.29		3.30 ± 1.57	
Enterprises	0.99 ± 0.68		2.35 ± 1.28		3.34 ± 1.52	
Workers	0.84 ± 0.67		2.20 ± 1.17		3.04 ± 1.46	
Farmers	0.81 ± 0.78		1.76 ± 1.28		2.56 ± 1.72	
Others	0.91 ± 0.70		2.30 ± 1.25		3.21 ± 1.51	
Family members of cardiac patients		0.001		<0.001		<0.001
Yes	1.00 ± 0.71		2.53 ± 1.17		3.54 ± 1.43	
No	0.94 ± 0.69		2.32 ± 1.29		3.26 ± 1.56	
Do not sure	0.82 ± 0.73		1.82 ± 1.30		2.64 ± 1.69	
Witnessed out-of-hospital cardiac arrest		0.153		<0.001		<0.001
Yes, and acting	0.89 ± 0.70		2.45 ± 1.20		3.34 ± 1.46	
Yes, but no acting	0.90 ± 0.72		2.59 ± 1.12		3.49 ± 1.37	
No	0.95 ± 0.70		2.25 ± 1.31		3.20 ± 1.60	
Trained in cardiopulmonary resuscitation		0.820		<0.001		<0.001
Yes	0.93 ± 0.68		2.73 ± 1.17		3.67 ± 1.41	
No	0.94 ± 0.71		2.19 ± 1.29		3.13 ± 1.58	

**Figure 3:**
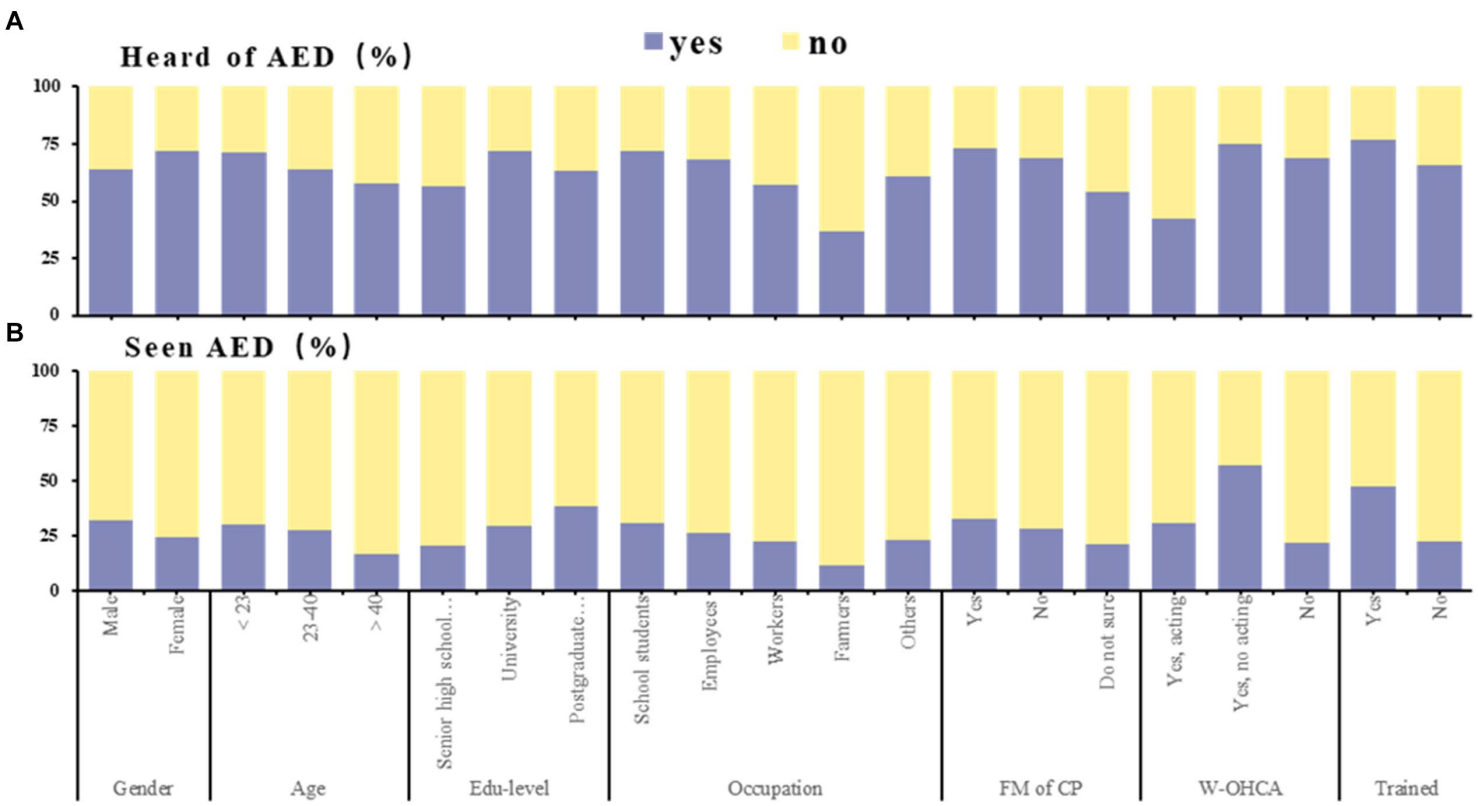
Awareness of automated external defibrillator among participants (*N* = 3,569). This figure shows the proportion of yes and no counts by sociodemographic characteristics in AED awareness. Association of sociodemographic characteristics with proportion of whether or not heard of AED **(A)**, whether or not seen AED **(B)**. AED, automated external defibrillator; FM of CP, family member of cardiac patient; W-OHCA, witnessed out-of-hospital cardiac arrest.

Most people had heard of AEDs (68.3%), but only a small number had seen AEDs (28.4%). The subgroup with the least heard of and seen AED remained farmers (36.6, 11.5%, *p <* 0.001), the subgroup with the most heard of AED was those who had participated in CPR training (76.8%, *p <* 0.001), and the subgroup with the most seen AED was those who had witnessed CA but did not participate in first aid (57.3%, *p <* 0.001; Supplementary Table S3; Figure 3).

Only a small number of people were willing to participate in first aid (22.6%), and the main reason for reluctance to participate in first aid was fear of imperfect operational skills (53.5%). The subgroup with the strongest desire for first aid was those who had been trained in CPR (44.9%, *p <* 0.001; Supplementary Table S4).

Among the basic factors, education level and family members of cardiac patients were significant predictors. Regarding operational knowledge, education level, family members of cardiac patients, and CPR training experience were significant predictors. Among the total knowledge, sex, education level, family members of cardiac patients, and CPR training experience were significant predictors (Supplementary Table S5).

Those who had experienced CPR training were more likely to have heard of (OR: 1.755) and seen (OR: 2.374) AEDs. Those who had seen OHCA were more likely to have seen AEDs (OR: 3.819). Families of cardiac patients were more likely to have heard of (OR: 2.063) and seen (OR: 1.483) AEDs. Compared to self-employed and retirees, school students were more likely to have heard of AEDs (OR: 1.479), and farmers were less likely to have heard of (OR: 0.422) and seen (OR: 0.489) AEDs. Male were less likely to have heard of AEDs (OR: 0.641) than female but were more likely to have seen AEDs (OR: 1.291; *p* < 0.05 for all; Supplementary Tables S6, S7).

A multivariate logistic regression equation was constructed by including CPR training experience, sex, age, and education level. The results showed that the willingness of participants who participated in CPR training to participate in first aid when witnessing OHCA increased by approximately 3 times (OR: 4.348), which was the most important factor. In addition, male (OR: 1.438), having witnessed OHCA (OR: 1.516), being a family member of cardiac patients (OR: 1.463), and education level of high school and below (OR: 1.612) also had a positive effect on increasing bystander CPR willingness. (*p* < 0.05 for all; Table 3).

**Table 3:** Multivariable logistic regression analysis between bystander CPR willingness and sociodemographic characteristics.

Variables	Bystander CPR willingness when witnessing out-of-hospital cardiac arrest		
	*Wald* χ^2^	OR	*95%*CI
Sex			
Male	17.692**	1.438	1.214–1.702
Female		1(ref)	
Age group, years	13.690*		
<23	11.476*	0.528	0.364–0.764
23–40	9.666*	0.627	0.468–0.842
>40		1(ref)	
Educational level	4.574		
High school or below	4.433*	1.612	1.034–2.514
Universities (including junior colleges)	2.412	1.391	0.917–2.109
Graduate degree or above		1(ref)	
Occupation	13.698*		
School students	8.237*	0.606	0.431–0.853
Enterprises	2.919	0.753	0.543–1.043
Workers	1.703	0.752	0.491–1.154
Farmers	7.231*	0.513	0.315–0.834
Others		1(ref)	
Family members of cardiac patients	5.872		
Yes	4.257*	1.463	1.019–2.100
No	5.827*	1.487	1.078–2.053
Do not sure		1(ref)	
Witnessed out-of-hospital cardiac arrest	16.338**		
Yes, and acting	0.553	0.879	0.626–1.234
Yes, but no acting	13.950**	1.516	1.219–1.886
No		1(ref)	
Trained in cardiopulmonary resuscitation			
Yes	239.137**	4.348	3.609–5.238
No		1(ref)	

*p < 0.05, **p < 0.001. OR, odds ratio; CI, confidential intervals; CPR, cardiopulmonary resuscitation.

## Discussion

The main findings of this study were as follows. First, the influencing factors of bystander’s CPR willingness included CPR training experience, bystander sex, age, education level, family members of heart patients and witnessed OHCA. Second, training experience was an important factor in improving bystanders’ CPR willingness, AED awareness and CA first aid knowledge. Third, farmers were a subgroup with the lowest AED awareness and cardiac arrest first aid knowledge.

Bystander CPR programs are not common in mainland China (25, 26), but this study found that CPR training can increase the willingness of bystanders to perform CPR. The survey results showed that the main reason for the unwillingness to perform bystander CPR when witnessing OHCA was the lack of confidence, and the improvement of CPR knowledge increased the confidence of bystanders to provide CPR (3). This was also consistent with the results of a study in South Korea (27). Additionally, this study found that males were more willing to perform bystander CPR than females, which was consistent with a study showing that male bystanders in the United States (28) and South Korea (29) were more willing to provide CPR. Furthermore, this study found that factors promoting the willingness of bystander CPR included older age (> 40 years), as is the case in the United Kingdom (14); the level of education in high school and below may be related to the fact that China began to popularize nine-year compulsory education at the beginning of the 21st century (30); knowing whether there were family members of patients with heart disease at home, and investigators who may care about their families have a stronger sense of social responsibility; and having witnessed OHCA, as is the case in the United Kingdom (14).

This study showed that nearly half of the people had never heard of AEDs, and less than 1/3 of the people had seen AEDs, especially farmers, who had the lowest awareness of AEDs in all occupations. Previous studies have also shown that AED configuration and use in China were rare (31), and compared with densely populated cities, rural AEDs were less configured. Even 52.3% of people with CPR training experience had not seen AEDs, indicating that China’s CPR training also needed to supplement AED use training tutorials. Meanwhile, Japan’s research on the outcome of OHCA patients showed that the effectiveness of nonprofessional defibrillation, only chest compression CPR and conventional CPR was weakened in turn (32). Therefore, it is very important for bystanders to immediately use AEDs and perform CPR when witnessing OHCA (14–23).

Although CPR training could significantly improve the total score of CA first aid knowledge, it had no obvious effect on the score of CA first aid basic knowledge. Most of the OHCAs in many countries (4), including China, occurred at home or residence (76.85%) (26), but the survival rate at home (7.8%) was less than half of that in public places (19%) (33). However, based on a survey of residents in central China, this study found that only the families of patients with heart disease could better understand that home was the most common place for OHCA. In contrast, those who had experienced CPR training or witnessed and acting on OHCA were more likely to believe that public places were the most common places for OHCA. British research suggests that first aid training is linked to increased knowledge of CPR (34). Therefore, Chinese residents need to improve their understanding of OHCA occurrence at home through first aid training. In terms of OHCA recognition, the training in this study did not show an obvious effect. Perhaps with the help of science and technology such as biosensors, the OHCA correct recognition rate could be improved more effectively (35). Therefore, in China, CPR training should pay more attention to supplementing CA first aid basic knowledge.

The influence of training experience on CA first aid operational knowledge was significant. It was crucial to witness OHCA patients making calls for the first time (36), and the shorter the response time of the emergency department was, the more effective the bystander CPR (1). In mainland China, prehospital emergency services were activated by only 120 calls (37). According to the survey, more than half of the subjects will call 120 for the first time, indicating that residents in central China had a strong sense of calling when witnessing OHCA. However, the position, frequency and depth of chest compressions also need to be trained and educated. The results of this study showed that bystanders who witnessed OHCA but did not act were more likely to correctly understand the location of chest compressions, which indicated that due attention should be given to increasing the chance of witnessing others performing chest compressions in CPR training. Meanwhile, people who received training were more aware of the chest compressions frequency, which confirmed the effectiveness of the training. In addition, the survey showed that bystanders who witnessed OHCA and acting could better remember the depth of chest compressions, which indicated that the depth of chest compressions in CPR training may be more impressed by the hands of the trainees. This survey showed that nearly half (50.9%) of the subjects had realized the importance of chest compressions alone, and studies had shown that chest compressions alone CPR training could help the public improve CPR skills more than conventional CPR training (32, 38).

From the perspective of the CA first aid knowledge correct rate, the knowledge of farmers was low, but the knowledge of family members of patients with heart disease and those who had participated in training was high (Figure 1). Specifically, there was almost no difference in basic knowledge, while operational knowledge was affected by family members of patients, witnessing OHCA, and training experience. Combined with the results of multiple linear regression analysis, education level and cardiac patient family members were significant influencing factors of first aid knowledge. However, in rural China, there is a lack of access to CPR-related knowledge, and more training is needed for CPR. A retrospective cohort study in Sweden also showed that there is still much room for training in OHCA first aid in rural areas (39).

## Limitations

First, due to the limitations of objective factors such as economy and epidemic situation, this study only conducts an online nonprobability random sampling survey on residents of a province in central China, so the research cannot be completely randomized stratified sampling. Second, because young college students in their 20s are more willing to participate in online surveys, the population ratio is imbalanced, and the research results have certain population limitations. Finally, considering that too much content of the questionnaire will affect the enthusiasm of the respondents to answer, the independent variables involved in this study are limited, and perhaps more positive factors affecting the willingness of bystanders to implement CPR have not been found. In the future, we will use a completely random stratified probability sampling method for face-to-face comprehensive in-depth investigation.

## Conclusion

In CA first aid knowledge, residents in central China had a higher awareness of recognition, call links and the importance of only chest compressions; the awareness of the OHCA most common location, location, frequency and depth of chest compressions was low. The AED allocation rate in a central province was low; furthermore, rural areas not only lacked AED allocation but also lacked first aid knowledge, so it was necessary to strengthen CPR training in rural areas. Training, witnessing OHCA experience and cardiac patient family members had a positive effect on bystander CPR willingness. Summarizing the characteristics of bystanders who were willing to first aid when witnessing OHCA was conducive to targeted CPR training, thereby improving the bystander CPR rate in mainland China.

## Data Availability

The raw data supporting the conclusions of this article will be made available by the authors, without undue reservation.
