# Supplementary material for: Training experience is an important factor affecting willingness for bystander CPR and awareness of AED: a survey of residents from a province in Central China in 2023
Source: Front Public Health. 2024 Sep 2;12:1459590. doi: 10.3389/fpubh.2024.1459590 (PMC11402821; doi:10.3389/fpubh.2024.1459590)
Supplement: Supplementary file 1 [file Table_1.docx]

# Table S1 Cardiac arrest first aid basic knowledge (N=3569)

| Variables | Where cardiac arrest occurs most often, N(%) | | | | Recognition, N(%) | |
| --- | --- | --- | --- | --- | --- | --- |
|  | Hospital | At home | Public places | Do not know | Yes | No |
| Total, N(%) | 252(7.1) | 1393(39.0) | 1414(39.6) | 510(14.3) | 1959(54.9) | 1610(45.1) |
| Sex |  |  |  |  |  |  |
| Male | 134(7.8) | 643(37.5) | 678(39.5) | 260(15.2) | 937(54.6) | 778(45.4) |
| Female | 118(6.4) | 750(40.4) | 736(39.7) | 250(13.5) | 1022(55.1) | 832(44.9) |
| χ² (*P* value) | 6.406(0.093) | | | | 0.086(0.770) | |
| Age group，years |  |  |  |  |  |  |
| <23 | 171(6.9) | 970(39.0) | 1055(42.5) | 288(11.6) | 1373(55.3) | 1111(44.7) |
| 23-40 | 64(9.9) | 267(41.4) | 223(34.6) | 91(14.1) | 358(55.5) | 287(44.5) |
| >40 | 17(3.9) | 156(35.4) | 136(30.9) | 131(29.8) | 228(51.8) | 212(48.2) |
| χ² (*P* value) | 120.458(＜0.001) | | | | 1.923(0.382) | |
| Educational level |  |  |  |  |  |  |
| High school or below | 60(8.5) | 247(34.9) | 229(32.3) | 172(24.3) | 344(48.6) | 364(51.4) |
| Universities (including junior colleges) | 177(6.6) | 1081(40.2) | 1121(41.6) | 312(11.6) | 1535(57.0) | 1156(43.0) |
| Graduate degree or above | 15(8.8) | 65(38.2) | 64(37.7) | 26(15.3) | 80(47.1) | 90(52.9) |
| χ² (*P* value) | 83.516(＜0.001) | | | | 20.601(＜0.001) | |
| Occupation |  |  |  |  |  |  |
| School students | 168(7.0) | 945(39.1) | 1022(42.3) | 279(11.6) | 1348(55.8) | 1066(44.2) |
| Enterprises | 27(7.0) | 173(45.1) | 141(36.7) | 43(11.2) | 209(54.4) | 175(45.6) |
| Workers | 5(3.5) | 46(32.4) | 59(41.6) | 32(22.5) | 73(51.4) | 69(48.6) |
| Farmers | 11(8.4) | 44(33.6) | 28(21.4) | 48(36.6) | 62(47.3) | 69(52.7) |
| Others | 41(8.2) | 185(37.2) | 164(32.9) | 108(21.7) | 267(53.6) | 231(46.4) |
| χ² (*P* value) | 119.359(＜0.001) | | | | 4.962(0.291) | |
| Family members of cardiac patients | | | | | | |
| Yes | 66(10.6) | 329(52.6) | 172(27.5) | 58(9.3) | 298(47.7) | 327(52.3) |
| No | 170(6.5) | 955(36.3) | 1144(43.5) | 361(13.7) | 1514(57.6) | 1116(42.4) |
| Do not sure | 16(5.1) | 109(34.7) | 98(31.2) | 91(29.0) | 147(46.8) | 167(53.2) |
| χ² (*P* value) | 147.696(＜0.001) | | | | 28.999(＜0.001) | |
| Witnessed out-of-hospital cardiac arrest | | | | | | |
| Yes, and acting | 30(13.3) | 82(36.5) | 91(40.4) | 22(9.8) | 118(52.4) | 107(47.6) |
| Yes, but no acting | 68(12.0) | 244(43.2) | 210(37.2) | 43(7.6) | 264(46.7) | 301(53.3) |
| No | 154(5.5) | 1067(38.4) | 1113(40.1) | 445(16.0) | 1577(56.7) | 1202(43.3) |
| χ² (*P* value) | 72.241(＜0.001) | | | | 19.624(＜0.001) | |
| Trained in cardiopulmonary resuscitation | | | | | | |
| Yes | 85(10.2) | 330(39.4) | 364(43.4) | 59(7.0) | 453(54.1) | 385(45.9) |
| No | 167(6.1) | 1063(38.9) | 1050(38.5) | 451(16.5) | 1506(55.1) | 1225(44.9) |
| χ² (*P* value) | 59.073(＜0.001) | | | | 0.306(0.580) | |
