# Supplementary material for: Training experience is an important factor affecting willingness for bystander CPR and awareness of AED: a survey of residents from a province in Central China in 2023
Source: Front Public Health. 2024 Sep 2;12:1459590. doi: 10.3389/fpubh.2024.1459590 (PMC11402821; doi:10.3389/fpubh.2024.1459590)
Supplement: Supplementary file 2 [file Table_2.docx]

# Table S2 Cardiac arrest first aid operational knowledge (N=3569)

| Variables | Phone and chest compression, N(%) | | Position of chest compression, N(%) | | Frequency of chest compression, N(%) | | Depth of chest compression, N(%) | | Importance of chest compression, N(%) | |
| --- | --- | --- | --- | --- | --- | --- | --- | --- | --- | --- |
|  | Yes | No | Yes | No | Yes | No | Yes | No | Yes | No |
| Total, N(%) | 2226(62.4) | 1343(37.6) | 1314(36.8) | 2255(63.2) | 1693(47.4) | 1876(52.6) | 1208(33.8) | 2361(66.2) | 1816(50.9) | 1753(49.1) |
| Sex |  |  |  |  |  |  |  |  |  |  |
| Male | 1025(59.8) | 690(40.2) | 658(38.4) | 1057(61.6) | 759(44.3) | 956(55.7) | 572(33.4) | 1143(66.6) | 881(51.4) | 834(48.6) |
| Female | 1201(64.8) | 653(35.2) | 656(35.4) | 1198(64.6) | 934(50.4) | 920(49.6) | 636(34.3) | 1218(65.7) | 935(50.4) | 919(49.6) |
| χ² (*P* value) | 9.536(0.002) | | 3.411(0.065) | | 13.387(＜0.001) | | 0.360(0.548) | | 0.314(0.575) | |
| Age group，years | | |  |  |  |  |  |  |  |  |
| <23 | 1537(61.9) | 947(38.1) | 923(37.2) | 1561(62.8) | 1245 (50.1) | 1239(49.9) | 885(35.6) | 1599(64.4) | 1241(50.0) | 1243(50.0) |
| 23-40 | 391(60.6) | 254(39.4) | 244(37.8) | 401(62.2) | 282(43.7) | 363(56.3) | 214(33.2) | 431(66.8) | 355(55.0) | 290(45.0) |
| >40 | 298(67.7) | 142(32.3) | 147(33.4) | 293(66.6) | 166(37.7) | 274(62.3) | 109(24.8) | 331(75.2) | 220(50.0) | 220(50.0) |
| χ² (*P* value) | 6.480(0.039) | | 2.605(0.272) | | 27.384(＜0.001) | | 19.829(＜0.001) | | 5.442(0.066) | |
| Educational level | | |  |  |  |  |  |  |  |  |
| High school and below | 423(59.7) | 285(40.3) | 242(34.2) | 486(65.8) | 278(39.3) | 430(60.7) | 194(27.4) | 514(72.6) | 343(48.4) | 365(51.6) |
| Universities (including junior colleges) | 1712(63.6) | 979(36.4) | 1004(37.3) | 1687(62.7) | 1342(49.9) | 1349(50.1) | 948(35.2) | 1743(64.8) | 1378(51.2) | 1313(48.8) |
| Graduate degree or above | 91(53.5) | 79(46.5) | 68(40.0) | 102(60.0) | 73(42.9) | 97(57.1) | 66(38.8) | 104(61.2) | 95(55.9) | 75(44.1) |
| χ² (*P* value) | 9.529(0.009) | | 3.136(0.208) | | 26.726(＜0.001) | | 17.312(＜0.001) | | 3.496(0.174) | |
| Occupation |  |  |  |  |  |  |  |  |  |  |
| School students | 1505(62.3) | 909(37.7) | 908(37.6) | 1506(62.4) | 1203(49.8) | 1211(50.2) | 843(34.9) | 1571(65.1) | 1208(50.0) | 1206(50.0) |
| Enterprises | 246(64.1) | 138(35.9) | 145(37.8) | 239(62.2) | 166(43.2) | 218(56.8) | 124(32.3) | 260(67.7) | 220(57.3) | 164(42.7) |
| Workers | 85(59.9) | 57(40.1) | 50(35.2) | 92(64.8) | 63(44.4) | 79(55.6) | 38(26.8) | 104(73.2) | 77(54.2) | 65(45.8) |
| Farmers | 70(53.4) | 61(46.6) | 32(24.4) | 99(75.6) | 39(29.8) | 92(70.2) | 30(22.9) | 101(77.1) | 59(45.0) | 72(55.0) |
| Others | 320(64.3) | 178(35.7) | 179(35.9) | 319(64.1) | 222(44.6) | 276(55.4) | 173(34.7) | 325(65.3) | 252(50.6) | 246(49.4) |
| χ² (*P* value) | 6.062(0.195) | | 9.771(0.044) | | 26.856(＜0.001) | | 12.031(0.017) | | 9.436(0.051) | |
| Family members of cardiac patients | | |  |  |  |  |  |  |  |  |
| Yes | 422(67.5) | 203(32.5) | 249(39.8) | 376(60.2) | 307(49.1) | 318(50.9) | 249(39.8) | 376(60.2) | 357(57.1) | 268(42.9) |
| No | 1641(62.4) | 989(37.6) | 983(37.4) | 1647(62.6) | 1280(48.7) | 1350(51.3) | 874(33.2) | 1756(66.8) | 1323(50.3) | 1307(49.7) |
| Do not sure | 163(51.9) | 151(48.1) | 82(26.1) | 232(73.9) | 106(33.8) | 208(66.2) | 85(27.1) | 229(72.9) | 136(43.3) | 176(56.7) |
| χ² (*P* value) | 21.699(＜0.001) | | 18.270(＜0.001) | | 25.875(＜0.001) | | 16.910(＜0.001) | | 17.282(＜0.001) | |
| Witnessed out-of-hospital cardiac arrest | | |  |  |  |  |  |  |  |  |
| Yes, and acting | 126(56.0) | 99(44.0) | 67(29.8) | 158(70.2) | 112(49.8) | 113(50.2) | 123(54.7) | 102(45.3) | 123(54.7) | 102(45.3) |
| Yes, but no acting | 328(58.1) | 237(41.9) | 244(43.2) | 321(56.8) | 274(48.5) | 291(51.5) | 230(40.7) | 335(59.3) | 388(68.7) | 177(31.3) |
| No | 1772(63.8) | 1007(36.2) | 1003(36.1) | 1776(63.9) | 1307(47.0) | 1472(53.0) | 855(30.8) | 1924(69.2) | 1305(47.0) | 1474(53.0) |
| χ² (*P* value) | 10.677(0.005) | | 15.272(＜0.001) | | 0.932(0.628) | | 67.213(＜0.001) | | 89.952(＜0.001) | |
| Trained in cardiopulmonary resuscitation | | |  | |  | |  | |  |  |
| Yes | 557(66.5) | 281(33.5) | 285(34.0) | 553(66.0) | 477(56.9) | 361(43.1) | 384(45.8) | 454(54.2) | 586(69.9) | 252(30.1) |
| No | 1669(61.1) | 1062(38.9) | 1029(37.7) | 1702(62.3) | 1216(44.5) | 1515(55.5) | 824(30.2) | 1907(69.8) | 1230(45.0) | 1501(55.0) |
| χ² (*P* value) | 7.834(0.005) | | 3.711(0.054) | | 39.513(＜0.001) | | 70.154(＜0.001) | | 158.951(＜0.001) | |
