# Supplementary material for: Training experience is an important factor affecting willingness for bystander CPR and awareness of AED: a survey of residents from a province in Central China in 2023
Source: Front Public Health. 2024 Sep 2;12:1459590. doi: 10.3389/fpubh.2024.1459590 (PMC11402821; doi:10.3389/fpubh.2024.1459590)
Supplement: Supplementary file 3 [file Table_3.docx]

# Table S3 Automated external defibrillator awareness(N=3569)

| Variables | Heard of AED, N(%) | | Seen AED, N(%) | |
| --- | --- | --- | --- | --- |
|  | Yes | No | Yes | No |
| Total, N(%) | 2436(68.3) | 1133(31.7) | 1012(28.4) | 2557(71.6) |
| Sex |  |  |  |  |
| Male | 1098(64.0) | 617(36.0) | 556(32.4) | 1159(67.6) |
| Female | 1338(72.2) | 516(27.8) | 456(24.6) | 1389(75.4) |
| χ² (*P* value) | 27.277(<0.001) | | 26.848(<0.001) | |
| Age group, years |  |  |  |  |
| <23 | 1773(71.4) | 711(28.6) | 759(30.6) | 1725(69.4) |
| 23-40 | 410(63.6) | 235(36.4) | 180(27.9) | 465(72.1) |
| >40 | 253(57.5) | 187(42.5) | 73(16.6) | 367(83.4) |
| χ² (*P* value) | 41.206(<0.001) | | 35.959(<0.001) | |
| Educational level |  |  |  |  |
| High school or below | 399(56.4) | 309(43.6) | 146(20.6) | 562(79.4) |
| Universities (including junior colleges) | 1929(71.7) | 762(28.3) | 800(29.7) | 1891(70.3) |
| Graduate degree or above | 108(63.5) | 62(36.5) | 66(38.8) | 104(61.2) |
| χ² (*P* value) | 62.614(<0.001) | | 32.514(<0.001) | |
| Occupation |  |  |  |  |
| School students | 1740(72.1) | 674(27.9) | 747(30.9) | 1667(69.1) |
| Enterprises | 263(68.5) | 121(31.5) | 101(26.3) | 283(73.7) |
| Workers | 81(57.0) | 61(43.0) | 32(22.5) | 110(77.5) |
| Farmers | 48(36.6) | 83(63.4) | 15(11.5) | 116(88.5) |
| Others | 304(61.0) | 194(39.0) | 117(23.5) | 381(76.5) |
| χ² (*P* value) | 96.920(<0.001) | | 35.352(<0.001) | |
| Family members of cardiac patients | |  |  |  |
| Yes | 458(73.3) | 167(26.7) | 206(33.0) | 419(67.0) |
| No | 1809(68.8) | 821(31.2) | 740(28.1) | 1890(71.9) |
| Do not sure | 169(53.8) | 145(46.2) | 66(21.0) | 248(79.0) |
| χ² (*P* value) | 37.811(<0.001) | | 14.904(0.001) | |
| Witnessed out-of-hospital cardiac arrest | |  |  |  |
| Yes, and acting | 95(42.2) | 130(57.8) | 70(31.1) | 155(68.9) |
| Yes, but no acting | 425(75.2) | 140(24.8) | 324(57.3) | 241(42.7) |
| No | 1916(68.9) | 863(31.1) | 618(22.2) | 2161(77.8) |
| χ² (*P* value) | 83.639 (<0.001) | | 285.762(<0.001) | |
| Trained in cardiopulmonary resuscitation | |  |  |  |
| Yes | 644(76.8) | 194(23.2) | 400(47.7) | 438(52.3) |
| No | 1792(65.6) | 939(34.4) | 612(22.4) | 2119(77.6) |
| χ² (*P* value) | 37.340(<0.001) | | 202.415(<0.001) | |
