# Supplementary material for: Training experience is an important factor affecting willingness for bystander CPR and awareness of AED: a survey of residents from a province in Central China in 2023
Source: Front Public Health. 2024 Sep 2;12:1459590. doi: 10.3389/fpubh.2024.1459590 (PMC11402821; doi:10.3389/fpubh.2024.1459590)
Supplement: Supplementary file 4 [file Table_4.docx]

# Table S4 Bystander CPR willingness when witnessing out-of-hospital cardiac arrest(N=3569)

| Variables | Yes, N(%) | No, most worried… N(%) | | | |
| --- | --- | --- | --- | --- | --- |
|  |  | The skill operation is not perfect | Disease contagion | Take responsibility | Do not sure |
| Total | 808(22.6) | 1909(53.5) | 113(3.2) | 160(4.5) | 579(16.2) |
| Sex |  |  |  |  |  |
| Male | 454(26.5) | 795(46.4) | 76(4.4) | 100(5.8) | 290(16.9) |
| Female | 354(19.1) | 1114(60.1) | 37(2.0) | 60(3.2) | 289(15.6) |
| χ² (*P* value) | 83.858(<0.001) | |  |  |  |
| Age group，years |  |  |  |  |  |
| <23 | 496(20.0) | 1449(58.3) | 84(3.4) | 101(4.1) | 354(14.2) |
| 23-40 | 171(26.5) | 279(43.2) | 25(3.9) | 45(7.0) | 125(19.4) |
| >40 | 141(32.1) | 181(41.1) | 4(0.9) | 14(3.2) | 100(22.7) |
| χ² (*P* value) | 106.808(<0.001) | |  |  |  |
| Educational level |  |  |  |  |  |
| High school or below | 199(28.1) | 309(43.6) | 23(3.3) | 32(4.5) | 145(20.5) |
| Universities | 570(21.2) | 1524(56.6) | 83(3.1) | 118(4.4) | 396(14.7) |
| Graduate degree  or above | 39(22.9) | 76(44.7) | 7(4.1) | 10(5.9) | 38(22.4) |
| χ² (*P* value) | 49.114(<0.001) | |  |  |  |
| Occupation |  |  |  |  |  |
| School students | 472(19.6) | 1418(58.7) | 70(2.9) | 104(4.3) | 350(14.5) |
| Enterprises | 100(26.0) | 171(44.5) | 18(4.7) | 19(5.0) | 76(19.8) |
| Workers | 44(31.0) | 60(42.3) | 2(1.4) | 7(4.9) | 29(20.4) |
| Farmers | 29(22.1) | 64(48.9) | 6(4.6) | 5(3.8) | 27(20.6) |
| Others | 163(32.7) | 196(39.4) | 17(3.4) | 25(5.0) | 97(19.5) |
| χ² (*P* value) | 99.896(<0.001) | |  |  |  |
| Family members of cardiac patients | |  |  |  |  |
| Yes | 165(26.4) | 327(52.3) | 29(4.6) | 34(5.5) | 70(11.2) |
| No | 586(22.3) | 1446(55.0) | 72(2.7) | 108(4.1) | 418(15.9) |
| Do not sure | 57(18.2) | 136(43.3) | 12(3.8) | 18(5.7) | 91(29.0) |
| χ² (*P* value) | 65.020(<0.001) | |  |  |  |
| Witnessed out-of-hospital cardiac arrest | | |  |  |  |
| Yes, and acting | 62(27.6) | 67(29.8) | 66(29.3) | 18(8.0) | 12(5.3) |
| Yes, but no acting | 206(36.4) | 269(47.6) | 28(5.0) | 28(5.0) | 34(6.0) |
| No | 540(19.4) | 1573(56.6) | 19(0.7) | 114(4.1) | 533(19.2) |
| χ² (*P* value) | 716.521(<0.001) | |  |  |  |
| Trained in cardiopulmonary resuscitation | | |  |  |  |
| Yes | 376(44.9) | 291(34.7) | 56(6.7) | 33(3.9) | 82(9.8) |
| No | 432(15.8) | 1618(59.2) | 57(2.1) | 127(4.7) | 497(18.2) |
| χ² (*P* value) | 382.585(<0.001) | |  |  |  |
