# Supplementary material for: Training experience is an important factor affecting willingness for bystander CPR and awareness of AED: a survey of residents from a province in Central China in 2023
Source: Front Public Health. 2024 Sep 2;12:1459590. doi: 10.3389/fpubh.2024.1459590 (PMC11402821; doi:10.3389/fpubh.2024.1459590)
Supplement: Supplementary file 5 [file Table_5.docx]

# Table S5 Multiple linear regression of cardiac arrest first aid knowledge scores

| Variables | Basic (2.00) | | Operational (5.00) | | Total (7.00) | |
| --- | --- | --- | --- | --- | --- | --- |
|  | **B**  ***95%*CI** | **t** | **B**  ***95%*CI** | **t** | **B**  ***95%*CI** | **t** |
| Sex |  |  |  |  | 0.155  0.054, 0.257 | 3.004* |
| Age group, years |  |  | -0.042  -0.121, 0.037 | -1.035 | -0.041  -0.138, 0.055 | -0.840 |
| Educational level | 0.063  0.011, 0.116 | 2.357* | 0.150  0.056, 0.244 | 3.125* | 0.214  0.099, 0.329 | 3.638** |
| Occupation | -0.008  -0.025, 0.009 | -0.911 | 0.001  -0.039, 0.040 | 0.035 | -0.008  -0.057, 0.040 | -0.335 |
| Family members  of cardiac patients  (ref. do not sure) | 0.085  0.039, 0.130 | 3.666** | 0.283  0.201, 0.364 | 6.805** | 0.369  0.269,0.469 | 7.252** |
| Witnessed out-of-hospital cardiac arrest  (ref. no) | |  | 0.063  -0.012,0.138 | 1.635 | 0.036  -0.057,0.128 | 0.760 |
| Trained in cardiopulmonary resuscitation (ref. no) | |  | 0.484  0.383,0.586 | 9.347** | 0.489  0.365,0.614 | 7.707** |

*p<0.05, **p<0.001

B, beta; CI, confidential intervals.

*p<0.05, **p<0.001

OR, odds ratio; CI, confidential intervals; CPR, cardiopulmonary resuscitation.
