# Supplementary material for: Training experience is an important factor affecting willingness for bystander CPR and awareness of AED: a survey of residents from a province in Central China in 2023
Source: Front Public Health. 2024 Sep 2;12:1459590. doi: 10.3389/fpubh.2024.1459590 (PMC11402821; doi:10.3389/fpubh.2024.1459590)
Supplement: Supplementary file 6 [file Table_6.docx]

# Table S6 Multivariable logistic regression analysis between AED awareness and sociodemographic characteristics

| Variables | Heard of AED | | |
| --- | --- | --- | --- |
|  | ***Wald* χ²** | **OR** | ***95%*CI** |
| Sex |  |  |  |
| Male | 35.580 ** | 0.641 | 0.554-0.742 |
| Female |  | 1(ref) |  |
| Age group, years | 0.166 |  |  |
| <23 | 0.166 | 0.934 | 0.672-1.298 |
| 23-40 | 0.063 | 0.966 | 0.739-1.264 |
| >40 |  | 1(ref) |  |
| Educational level | 8.561* |  |  |
| High school or below | ＜0.001 | 0.998 | 0.684-1.455 |
| Universities | 2.770 | 1.345 | 0.949-1.907 |
| Graduate degree or above | | 1(ref) |  |
| Occupation | 29.386** |  |  |
| School students | 6.511* | 1.479 | 1.095-1.997 |
| Enterprises | 2.503 | 1.272 | 0.944-1.712 |
| Workers | 0.223 | 0.910 | 0.617-1.344 |
| Farmers | 16.660** | 0.422 | 0.279-0.638 |
| Others |  | 1(ref) |  |
| Family members of cardiac patients | 24.224** | |  |
| Yes | 23.414** | 2.063 | 1.539-2.767 |
| No | 16.651** | 1.689 | 1.320-2.160 |
| Do not sure |  | 1(ref) |  |
| Trained in cardiopulmonary resuscitation | | |  |
| Yes | 35.703** | 1.755 | 1.459-2.110 |
| No |  | 1(ref) |  |

*p<0.05, **p<0.001

OR, odds ratio; CI, confidential intervals; AED, automated external defibrillator.
