# Supplementary material for: Training experience is an important factor affecting willingness for bystander CPR and awareness of AED: a survey of residents from a province in Central China in 2023
Source: Front Public Health. 2024 Sep 2;12:1459590. doi: 10.3389/fpubh.2024.1459590 (PMC11402821; doi:10.3389/fpubh.2024.1459590)
Supplement: Supplementary file 7 [file Table_7.docx]

# Table S7 Multivariable logistic regression analysis between AED awareness and sociodemographic characteristics

| Variables | Seen AED | | |
| --- | --- | --- | --- |
|  | ***Wald* χ²** | **OR** | ***95%*CI** |
| Sex |  |  |  |
| Male | 10.123* | 1.291 | 1.103-1.510 |
| Female |  | 1(ref) |  |
| Age group, years | 3.036 |  |  |
| <23 | 2.073 | 1.334 | 0.901-1.974 |
| 23-40 | 2.845 | 1.333 | 0.955-1.862 |
| >40 |  | 1(ref) |  |
| Educational level | 11.307* |  |  |
| High school or below | 11.195* | 0.498 | 0.331-0.749 |
| Universities | 5.928* | 0.635 | 0.440-0.915 |
| Graduate degree or above | | 1(ref) |  |
| Occupation | 9.158 |  |  |
| School students | 2.437 | 1.306 | 0.934-1.827 |
| Enterprises | 0.065 | 1.045 | 0.744-1.468 |
| Workers | 0.210 | 0.892 | 0.548-1.453 |
| Farmers | 5.098* | 0.489 | 0.263-0.910 |
| Others |  | 1(ref) |  |
| Family members of cardiac patients | 5.174 |  |  |
| Yes | 5.118* | 1.483 | 1.054-2.085 |
| No | 3.829 | 1.353 | 1.000-1.832 |
| Do not sure |  | 1(ref) |  |
| Witnessed OHCA | 169.535** |  |  |
| Yes, and acting | 0.668 | 1.140 | 0.833-1.561 |
| Yes, but no acting | 166.888** | 3.819 | 3.117-4.680 |
| No |  | 1(ref) |  |
| Trained in cardiopulmonary resuscitation | |  |  |
| Yes | 91.633** | 2.374 | 1.989-2.833 |
| No |  | 1(ref) |  |

*p<0.05, **p<0.001

OR, odds ratio; CI, confidential intervals; AED, automated external defibrillator.
